# Mimicking the Natural Basement Membrane for Advanced Tissue Engineering

**DOI:** 10.1021/acs.biomac.2c00402

**Published:** 2022-07-15

**Authors:** Puja Jain, Sebastian Bernhard Rauer, Martin Möller, Smriti Singh

**Affiliations:** † DWI-Leibniz-Institute for Interactive Materials e.V, Aachen 52074, Germany; ‡ Chemical Process Engineering, RWTH Aachen University, Aachen 52074, Germany; § Max-Planck-Institute for Medical Research, Heidelberg 69028, Germany

## Abstract

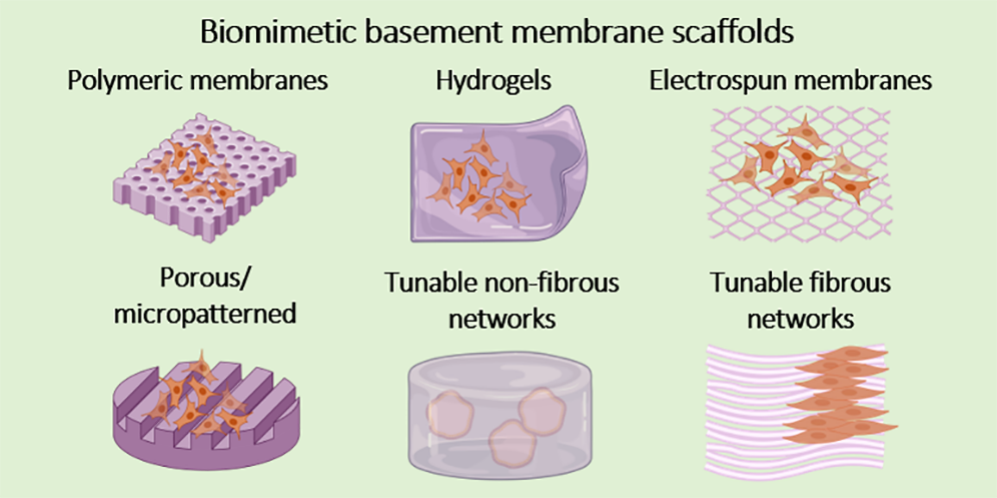

Advancements in the field of tissue engineering have led to the elucidation of physical and chemical characteristics of physiological basement membranes (BM) as specialized forms of the extracellular matrix. Efforts to recapitulate the intricate structure and biological composition of the BM have encountered various advancements due to its impact on cell fate, function, and regulation. More attention has been paid to synthesizing biocompatible and biofunctional fibrillar scaffolds that closely mimic the natural BM. Specific modifications in biomimetic BM have paved the way for the development of *in vitro* models like alveolar-capillary barrier, airway models, skin, blood-brain barrier, kidney barrier, and metastatic models, which can be used for personalized drug screening, understanding physiological and pathological pathways, and tissue implants. In this Review, we focus on the structure, composition, and functions of *in vivo* BM and the ongoing efforts to mimic it synthetically. Light has been shed on the advantages and limitations of various forms of biomimetic BM scaffolds including porous polymeric membranes, hydrogels, and electrospun membranes This Review further elaborates and justifies the significance of BM mimics in tissue engineering, in particular in the development of *in vitro* organ model systems.

## Introduction

**Figure 1:**
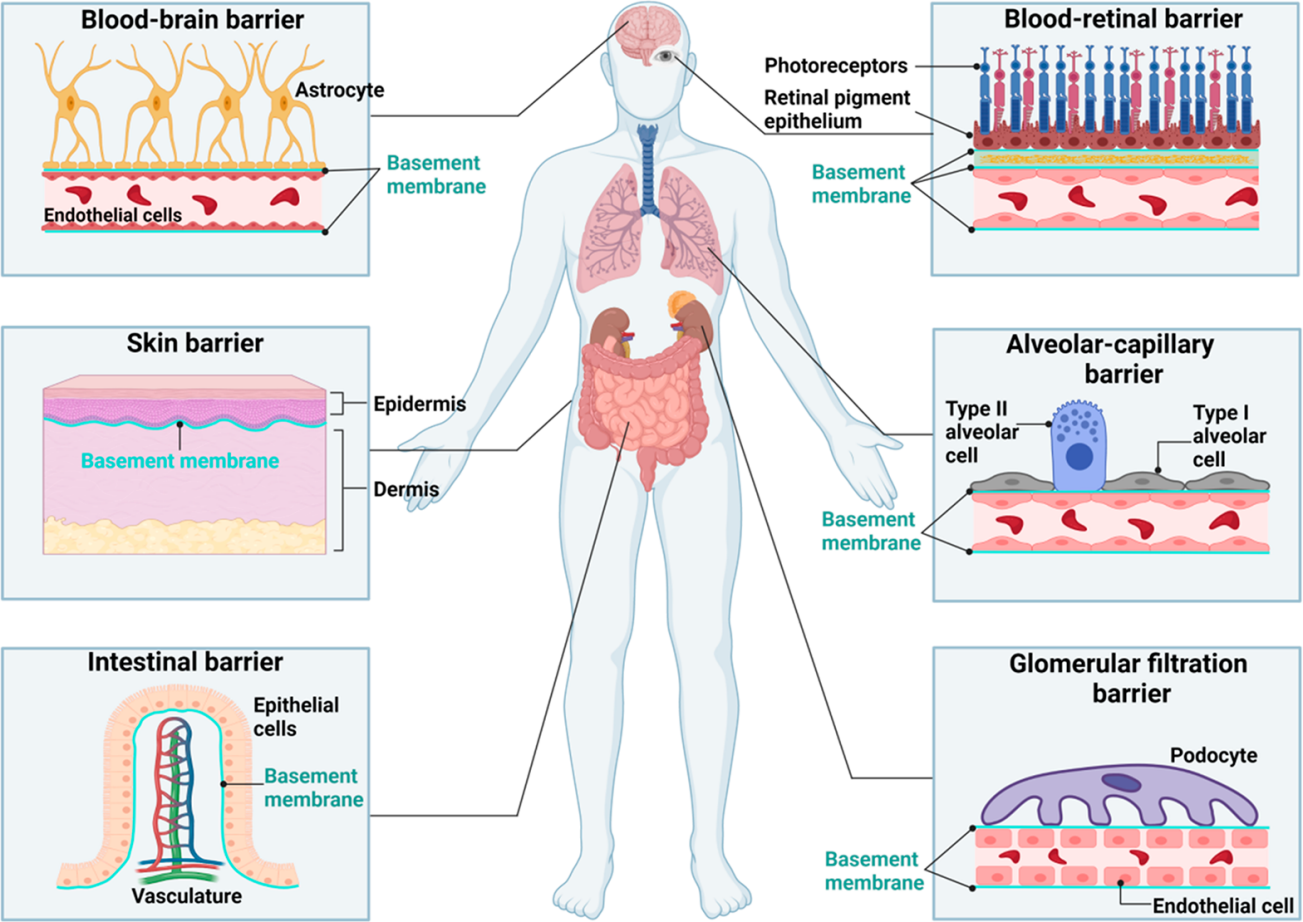
Basement membrane location: Basement membrane (BM) is ubiquitous in the human body and is located adjacent to the epithelium, endothelium, and parenchymal cells including muscle, adipose as well as nerve cells. It is involved in many vital physiological processes and is found in many organ barriers including the brain, retina, kidney, intestine, and lung. The schematic displays examples of some of the vital organs where the BM can be found. This includes the underlying areas of the epithelium and endothelium, where it supports and physically separates the different cellular layers. Created with Biorender.com

Cells reside in a tissue environment that is primarily composed of water, proteoglycans (glyocaminoglycans), and proteins, such as collagen, elastin, fibronectin, and laminin.^1^ This noncellular microenvironment is termed as the extracellular matrix (ECM) and plays a major role in controlling cellular behavior, tissue formation, and homeostasis. There exists a variation in ECM at different tissue locations due to the different compositions, combinations, and arrangements of proteins and glycans that make up the ECM.^2^ In 1857, the term basement membrane (BM) was first used by Robert Todd and William Bowman to describe the specialized ECM membrane on which epithelial cells rest as a “continuous basement membrane of excessive tenuity, apparently identical with that which supports the epithelium of mucous membranes”.^3^ The BM is located basolateral to the epithelial and endothelial cell layers and surrounds peripheral nerve axons, adipose, and muscle cells.^4^ It is ubiquitous and forms a continuous sheath around all vital organs including the cardiovascular, nervous, respiratory, excretory, digestive, and integumentary systems,^5−14^ as represented in Figure 1. Functionally, this dynamic structure is involved in regulating and maintaining biochemical signals between cells and their surrounding tissues, apart from providing physical support.^15−19^ BMs also maintain organ shape and size and their significance is further observed in the development of diseases due to genetic mutations in BM genes.^20^ Mutations in genes that code for the collagen IV network including COL4A3, COL4A4, COL4A5 can lead to Alport Syndrome or thin BM nephropathy (TBMN) that affect the kidney filtration barrier.^21^ Additionally, defects in genes coding CD151 are associated with defects in the glomerular BM, and defects in the Lmγ1 gene leads to embryonic death associated with nondeveloped BM.^22,23^ Moreover, defects in BM regeneration or development have also been observed in cases of corneal stromal fibrosis and epidermolysis bullosa.^24,25^

The above-mentioned vital functions deem it necessary to design scaffolds that closely resemble the structural, mechanical, and functional characteristics of the native BM. Furthermore, the relevance of an appropriate scaffold design is described in a vast array of research that highlight the influence of biophysical and biochemical signals on cellular processes such as proliferation, migration, differentiation, and gene expression.^26−30^ Scaffold biophysical cues include tensile modulus, pore size, roughness, and topology, whereas biochemical cues include growth factors, cell adhesion ligands, hormones, and other molecules that influence cell behavior.^31^ Discrepancies observed in the interaction of cells between fibers and flat hydrogel surfaces are highlighted in the works of Baker and colleagues.^32^ Their experimental results, involving the interaction of NIH 3T3 fibroblast cells with methacrylated dextran (DexMa) in the form of electrospun fibers and hydrogel surfaces, implicate the sensitivity of cells toward the scaffold’s architecture. An opposite behavior is observed on the fiber and hydrogel scaffolds where cell spreading is prominent on stiff hydrogels as compared with stiff fibers and vice versa. Similar differences in the expression of αSMA by endothelial cells on nonwoven electrospun meshes and PET membranes were observed by Jain et.al.^33^ This illustrates the influence of scaffold structure and property on cell behavior when used to mimic ECM like materials such as the BM. Furthermore, Slater et al. demonstrated confluent monolayer formation of cells on the electrospun membranes as opposed to that on hydrogel systems with embedded adhesive ligands and Matrigel.^34^

To reach the goal of a native-like scaffold, a wide variety of different biomaterials have been synthesized and manufactured, which feature typical BM properties such as intricate fibrillar architecture, the viscoelastic mechanical properties, the adhesive sequences, the dynamic enzyme-induced nature as well as the possibility of controlled storage and release of bioactive substances such as growth factors.^33,35−43^ These biomaterials are mostly fabricated from natural and synthetic polymer systems and try to combine multiple BM properties that are tailored toward the needs of a desired tissue construct or a region of interest.^44^ Scaffolds are used to construct *in vitro* models that are valuable research tools for unraveling fundamental biological processes involved in tissue homeostasis and disease development as well as serving as industrial platforms for drug screening.^5,6,8−14,45^ Furthermore, despite the success of animal models as an invaluable source of scientific knowledge, animal models are often limited in translation to human biology and response.^46,47^ Additionally, the three R’s principle when conducting animal studies (reduction, replacement, and refinement) abide by animal ethics and do promote the use of other systems such as simulation or *in vitro* models when possible.^48,49^ Therefore, the application of *in vitro* models and scaffolds might not only uncover scientific knowledge and save patient lives but also reduce the necessity of animal studies.

The importance of mimicking the BM has been highlighted in this Review by elaborating on its structure, assembly, function, and location in the human body. We provide the readers an understanding of the various forms of available BM mimics from naturally derived to synthetic materials as well as their strengths and weaknesses. This Review further aims to help understand key points required to construct appropriate BM scaffolds that closely represent their *in vivo* counterparts.

## Structure and Assembly of Natural BM

The BM comprises basal lamina (subdivided into lamina lucida\rara and lamina densa) and the reticular lamina. Closer to the parenchymal cell layer with an average thickness of 27 nm is the lamina lucida, and underlying this layer, closer to the connective tissue, is the dense fibrillar lamina densa with an average thickness of 53 nm. The reticular lamina is observed to be structurally similar to the loose interconnective tissue. Recently, the basal lamina is also referred to as the BM and the words are used interchangeably.^50,51^ BM is considered a thin (50–300 nm) fibrillar layer resembling the ECM underlying the parenchymal cell layers that separate it from the connective tissue.^52,53^

**Figure 2:**
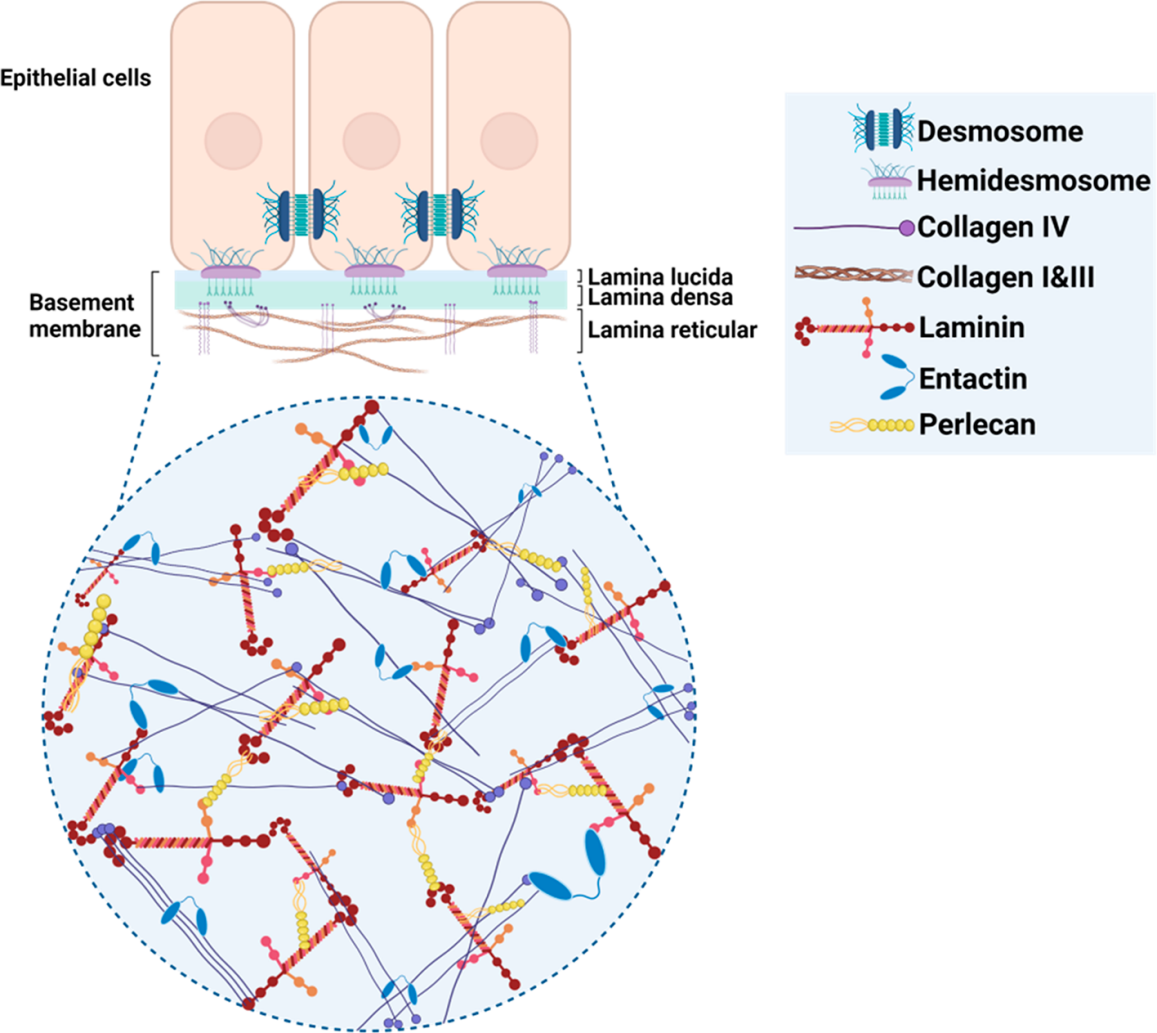
Structural feature of basement membrane: A dense fibrillar layer, primarily located underneath the epithelial cells, the lamina lucida, and densa together with the lamina reticular correspond to the basement membrane. At the molecular level, it is primarily composed of dynamic laminin and a network of collagen IV that are bound together by entactin (nidogen) along with perlecan to form a network of supporting ECM for cellular layers. Created with Biorender.com.

Molecularly, BM is composed of varying combinations and isoforms of four principle biomolecules that include collagen IV, laminin, nidogen, and perlecan at various tissue locations (Figure 2).^54^ Other molecules include agrin, fibronectin, fibrinogen, and collagen type XV and type XVIII.^55^ The highly cross-linked collagen together with the more dynamic noncovalent laminin isoform network provides mechanical stability to the BM. Nidogen, also known as entactin, binds the collagen IV and laminin and also binds to perlecan, fibronectin, and fibrinogen.^56,57^ Type IV collagen in mammals is a combination of 6 distinct α polypeptide chains (α1-α6). The α chains have three domains: amino-terminal 7S domain, a middle triple-helical domain (1400 amino acid), and a noncollagenous carboxy-terminal globular domain (NC-1) (230 amino acid). Repetitive units of Gly-X-Y are found in the triple helical\collagen and 7S domains. The triple-helical domain has 22 interruptions that administer flexibility to it. The 6-α chains are 50–70% homologous at the amino acid level and differ in their NC1 domain. Cells produce collagen IV in the form of protomers (heterotrimers consisting of three α chain combinations). Different protomer combinations of collagen IV contribute to 50% of the BM found at different locations.^51,58,59^ Laminin is a heterotrimeric protein derived from genes that code for α (1–5), β (1–3), and γ (1–3) chains. The average size of the α chain is 400 kDa, and β and γ are 200 kDa.^60^ Constituting the second major component of the BM, the laminin isoforms (a combination of the different α, β, and γ chains) resemble a three-pronged-fork that stems from six domains of the chain. The C-terminal of the α, β, and γ chains form the handle (consist of I and II domains of each chain), whereas the N-termini short arms of the chains (consisting of III, IV, V, and VI domains of each chain) form the prongs.^61^ Nidogen, also known as entactin, is a glycoprotein that makes up nearly 3% of the BM. Transcription of the genes NID1 and NID2 lead to the formation of Nidogen1 (30 nm long) and Nidogen2 (40 nm long) respectively.^62^ The two forms are prevalent in the differently localized BM. Nidogen binds to collagen, laminin, fibronectin, and perlecan.^51,59,63^ Perlecan is a 450 kDa heparan sulfate glycoprotein that is ubiquitous in the BM and has binding sites on collagen, nidogen, and laminin. Structurally perlecan consists of five domains (I–V) akin to a pearl on a string arrangement.^51,59,64^

The varying composition and isoforms of the above-mentioned components trigger specificity to the BM that is prevalent at its different anatomical locations, Table 1. Despite differences in molecular isoforms of the principle components at various tissue locations, they share a similar strategy of self-assembly.^56^ Protomers of collagen IV and laminin heterotrimers are assembled in the Golgi apparatus inside the cell along with other single molecules such as nidogen and perlecan. Secretory vesicles transport these molecules to the extracellular environment, where laminins assemble on the cell surface receptors via binding motifs. Secreted collagen IV protomers form networks with the assembled laminin through nidogen and perlecan.^55,56^ The cascade of intracellular production and secretion followed by extracellular self-assembly leads to BM formation.

**Table 1:** Varying Components of Naturally Occurring BM Based on Tissue Location

tissue	collagen	laminin	nidogen	others	ref
blood-brain barrier BM	collagenIV	laminin211, laminin411, laminin511	nidogen-1, -2	perlecan, agrin, fibronectin	(65)
skin BM	collagenIV, collagenVII,	laminin511, laminin411, laminin322	nidogen-1, -2	perlecan, agrin, fibrillar	(66)
intestinal barrier BM	collagenIV	laminin111, laminin511, laminin332	nidogen-1, -2	perlecan, agrin, fibulin	(67,68)
corneal BM	collagenIV, collagenVII, collagenXII, collagenXVII, collagenXVIII,	laminin311, laminin333, laminin411, laminin511	nidogen-1, -2	perlecan, fibronectin	(11)
alveolar-capillary BM	collagenIV, collagenVIII	laminin411, laminin511	nidogen-1, -2	perlecan, agrin, fibronectin	(69,70)
glomerular BM	collagenIV	laminin221, laminin521	nidogen-1, -2	agrin	(7,71)

## Properties and Function of the Natural BM

Cells adhere to the underlying BM by an interaction between their receptor proteins such as integrins to adhesion motifs on the collagen and laminin networks, as seen in Figure 2.^1,72^ It provides physical support to the overlying epithelial and endothelial tissues and also acts as an interface to the interstitial stroma.^73^ The complexity of the BM structure enables permeation and diffusion of selective molecules which impart filtration properties including the glomerular kidney BM responsible to filter blood.^74^ Interaction of heparan binding growth factors including fibroblast growth factor (FGF), vascular endothelial growth factor (VEGF), and platelet-derived growth factor (PDGF) are achieved via perlecan and its heparan sulfate chain in the BM.^75−77^ Storage and release of specific growth factors, ions, and hormones highlight its involvement in tissue development and remodeling processes.^78−83^ For example, sequestered VEGF bound to heparan sulfate (perlecan) in the BM is involved in the development of new vasculature during an injury.^84−86^ Moreover, apart from the provision of cell adhesion sites, the interaction of cell receptors to varying configurations of ligands on the underlying BM surface triggers a cascade of intracellular reactions responsible for the altered behavior of cells in specific organs.^87,88^ In addition to these biochemical cues, cell response is also controlled by biophysical cues such as BM architecture and elastic modulus,^89−91^Table 2. Substrate topography and mechanical properties modulate cell morphology, proliferation, migration, genetic expression, and activation of intracellular signaling pathways.^92−95^ A wide variety of research involved in the field of mechanotransduction reveals the importance of understanding the structural properties of the BM.^96,97^ Modulus measurement using AFM (atomic force microscopy), micro and nanoindentation have enabled researchers to investigate the young’s modulus of various BM at different tissue locations.^98−100^ Modulus measurements in the range of 1–3 MPa for chick inner limiting membrane and mouse retinal inner limiting membrane between 3.8 and 4.1 MPa has been reported.^4^ However, Young’s modulus is not universal and has varying values based on species age as well as tissue location.^101^ Besides these mechanical BM properties, the structural features of BMs including thickness, porosity, and pore size significantly impact cellular behavior and function.^102−105^ Transmission electron microscopy and scanning electron microscopy of the BM have revealed a fibrous structure with interconnected pores.^106−108^ The undulated surface of the BM is characterized by fiber diameters in the range of 30–400 nm along with pores from 10–150 nm.^108−110^ Due to its network structure, it also behaves like a physical barrier between the overlying cells and the underlying connective tissue, thereby controlling the movement of solutes and cells across it.^56,57^ The arrangement of the BM structure is such that pores of an average size of 50 nm allow passive diffusion of small solutes.^52^ Despite the small pore size, cells can transmigrate through the BM during an immune response or a normal tissue development event. Extensive studies in the literature have investigated cellular transmigration through the BM.^111−116^

**Table 2:** Biophysical Properties of Naturally Occurring BM Vary Based on Species and Organ

species, tissue	Young’s modulus [kPa]	ultimate tensile strength [MPa]	pore size [nm]	thickness [μm]	ref
human, anterior BM of cornea	7.5 ± 4.2	3.81 ± 0.4	92	<0.5	(98,124)
human, Descemet’s membrane of cornea	50 ± 17.8	1.72 ± 0.19	38	>5	(98,101)
mouse, Matrigel	0.45	-	-	1000	(100)
cat, lens capsule BM	820	1.7	-	61	(125)
rabbit, anterior BM of the cornea	4.5 ± 1.2	3.83 ± 0.91	-	-	(126,127)
rabbit, renal tubule	500	0.5	-	0.26	(128)

In addition to the mentioned functions of the BM, its significance is further enhanced by the repercussion endured in the events of genetic abnormalities that lead to disorders such as Alport syndrome and Knoblach syndrome due to mutations in the type IV and XVIII collagen molecules, respectively.^117,118^ These mutations affect the mechanical properties of the BM including the thickness and its stiffness. In the case of Alport syndrome, the collagen IV network is not highly cross-linked leading to a deformed pore and unstable thicker BM at the glomerular filtration barrier.^21^ Mouse models representing Alport syndrome had a 30% lower Young’s modulus compared with control mice despite a higher collagen IV content.^119^ Similar thickening of vascular BM is observed during Alzheimer’s disease due to the deposition of collagen IV and amyloid-β-accumulation.^120^ Retinal vascular BM was observed to be thicker and softer in human patients with diabetes (1.5 kPa) compared with nondiabetic patients (5.1 kPa).^121^ Moreover, an age-related increase in BM thickness has been supported by many studies.^15^ Changes in biochemical and biophysical properties of BM are observed during aging and disease progression as mentioned above, and their causes need to be investigated further.

In brief, the BM provides physical support and adhesion receptors to intermediate cells and a microenvironment rich in growth factors that influence cellular behavior such as proliferation, differentiation, and regenerative processes such as tissue repair. Research evidence also suggests the influence of structural and molecular diversity of the BM on cellular interactions, morphogenesis, and transmigration during immune surveillance as well as metastasis which further amplifies the need to incorporate such features in BM mimics.^122,123^

## Recapitulating the BM

**Figure 3:**
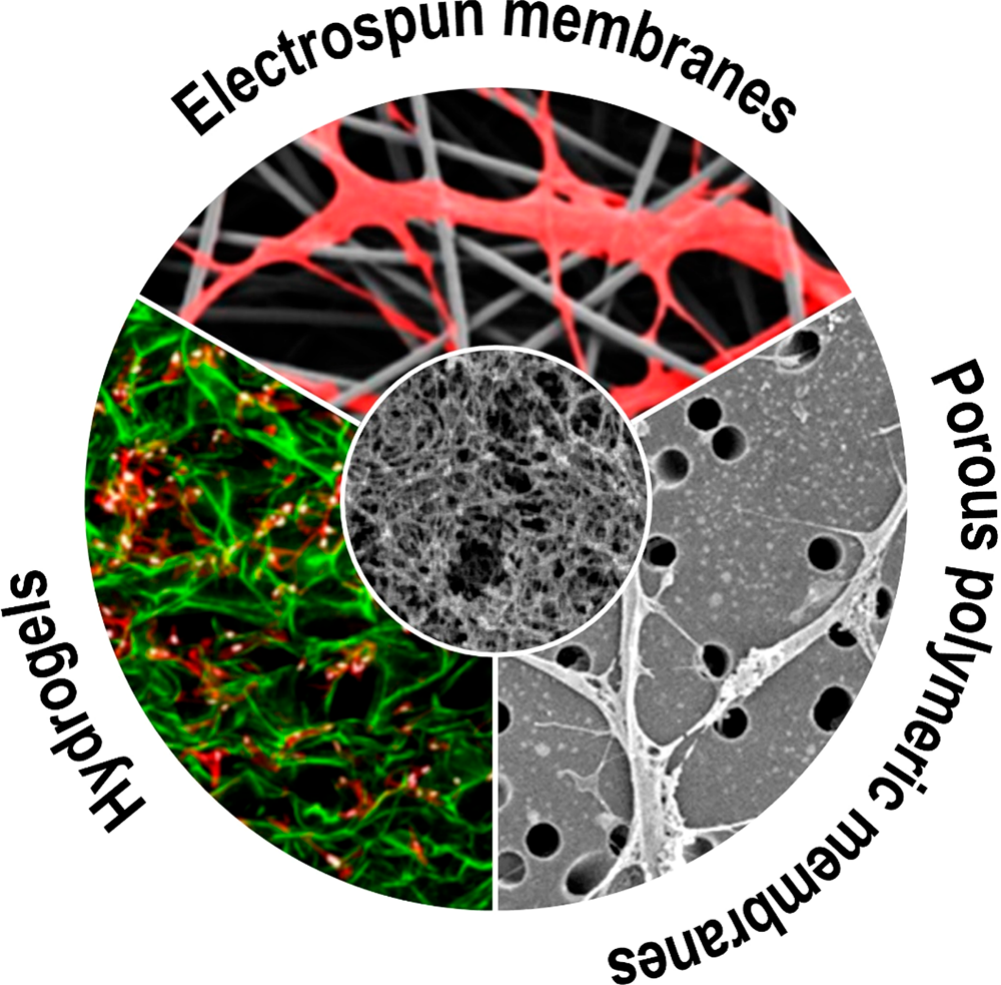
Structural resemblance of native basement membrane with synthetic mimics: Schematic represents the structural resemblances and differences observed by comparison of the scanning electron microscope (SEM) and confocal images between the Matrigel BM (center inset); adapted with permission from ref (138). Copyright 2006 Gelain et al. http://creativecommons.org/licenses/by/4.0/ (changes were made) and commonly used synthetic platforms including electrospun membranes (top); adapted with permission from ref (139). Copyright 2019 Ura, Daniel P et al. http://creativecommons.org/licenses/by/4.0/ (changes were made) and the hydrogels (bottom left); adapted with permission from ref (140). Copyright 2015 Elizabeth A. Wahl et al. https://creativecommons.org/licenses/by/3.0/ (changes were made) and track-etched membranes such as porous poly(ethylene terephthalate) (PET) (bottom right); adapted with permission from ref (141). Copyright 2018 Julian H. George et al. http://creativecommons.org/licenses/by/4.0/ (changes were made). Electrospinning allows alteration of membrane properties including thickness, fiber diameter, pore size, and fiber density, topography as well as mechanical properties. Synthesis of hydrogels with high water content also enables the formation of nonfibrous scaffolds with varying mechanical properties based on cross-linking density. However, despite the ease of handling and reproducibility, the use of porous polymeric membranes as synthetic BM mimics is controversial based on wide mechanical and topographical differences from the native BM.

Over the years, biomaterial research has produced an impressive amount of different synthetic material systems that can closely resemble a broad spectrum of essential native BM characteristics.^129^ These BM mimics reach from simple porous polymeric membranes to highly structured electrospun fiber mats as well as 3D hydrogel systems (Figure 3) which can be produced from both natural and synthetic polymers or their arbitrary combinations. Considering their synthetic nature, engineered biomaterials can not only reduce the need for animal-derived products but also provide the opportunity to precisely tailor BM scaffolds to the biophysical and biochemical needs of a specific tissue region.^130,131^ In this regard, a wide variety of macro- and microarchitectures could be realized based on different fabrication techniques, while advances in chemistry such as controlled peptide synthesis or click chemistry enable scaffold functionalization and adjustment of possible mechanical features.^132−137^ These new synthetic BMs scaffolds not only replicate naturally derived BM but also are promising systems to improve on some of their limitations, including reproducibility and stable mechanical properties, and to study the effects of isolated BM properties on cellular behavior.

## Porous Polymeric Membranes as BM Mimics

**Figure 4:**
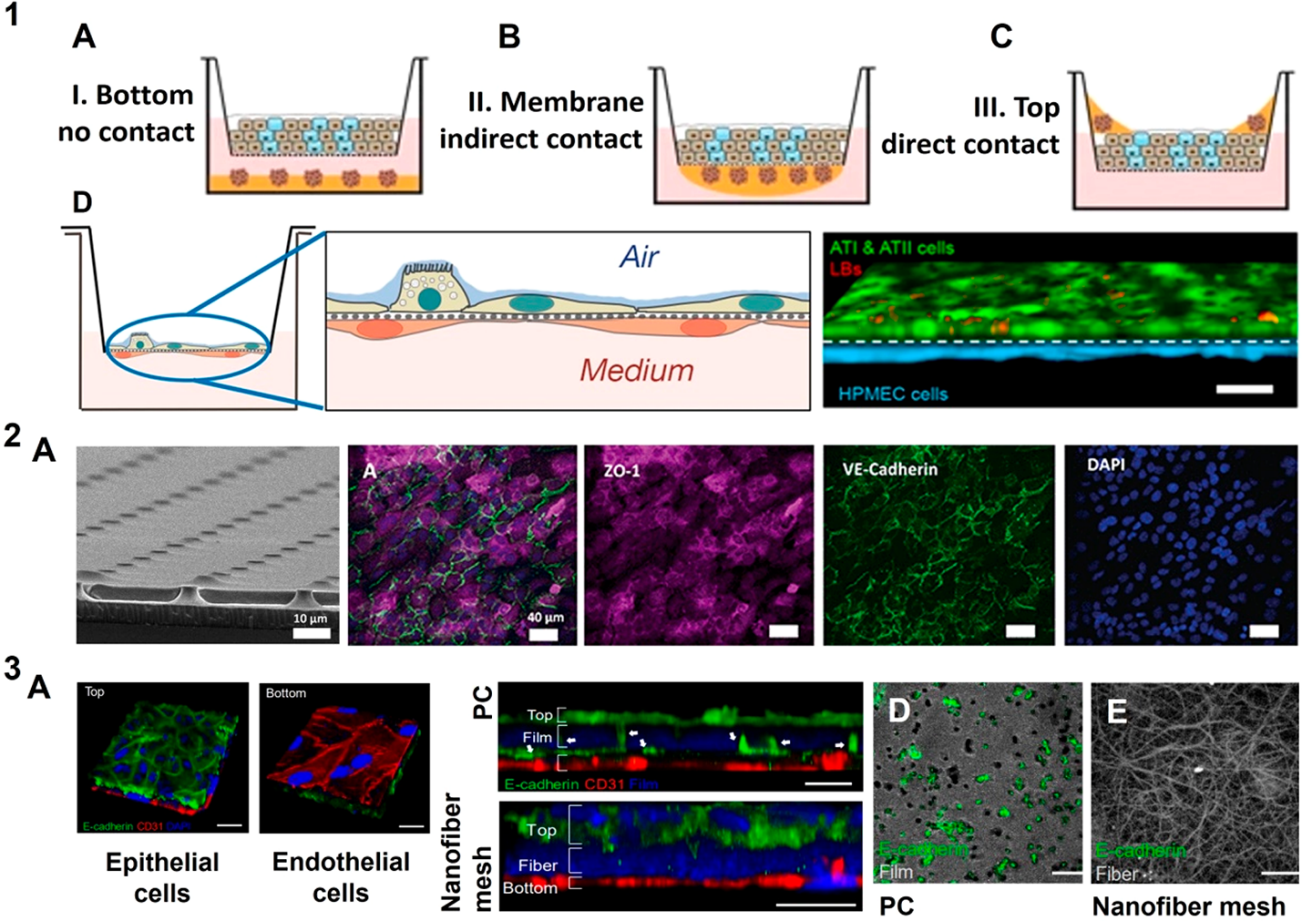
Porous polymeric membranes as basement membrane mimic: Depiction of possible combinations regarding cell coculture models using mechanically stable dense porous polymeric membranes: (1A) noncontact cocultures of two spatially separated cell types, (1B) indirect cocultures of two different cell types in contact with the same membrane, and (1C) direct mixture of two different cell types on top of the same membrane; adapted with permission from ref (164). Copyright 2017 Qiaozhi Lu et al. http://creativecommons.org/licenses/by/4.0/ (changes were made). (1D) Alveolar-capillary barrier models on permeable 0.4 μm pore size polyester membranes which can be introduced to an air–liquid interface represents the ease of handling coculture barriers on such mechanically robust platforms; adapted with permission from ref (165). Copyright 2021 Shinjini Chakraborty et al. http://creativecommons.org/licenses/by/4.0/ (changes were made). (2A) Mechanically tunable PDMS membranes and their reduced thickness to 2 μm were used to represent blood-brain barrier, by mono/coculture of endothelial cells and astrocytes where endothelial cells are stained for peripheral tight junctions ZO-1(red), adherens junction protein VE-cadherin (green); reprinted with permission from ref (144). Copyright 2020 Royal Society of Chemistry https://creativecommons.org/licenses/by/3.0/ (changes were made). Limitations of porous polymeric membranes are elucidated: (3A) Alveolar-capillary barrier on nanofiber PCL mesh and their cross-section on PC and nanofiber mesh which shows epithelial (green) transmigration toward the endothelial cells across a PC membrane (blue) with 3 μm pores compared to intact alveolar epithelial and endothelial cocultures on a nanofiber mesh, where the epithelial protrusion (green) is clearly seen on opposite side of (3D) PC membrane compared to the (3E) nanofiber mesh; reprinted with permission from ref (41). Copyright 2017 American Chemical Society (changes were made).

Biocompatible polymers such as poly(dimethylsiloxane) (PDMS), poly(carbonate) (PC), and poly(ethylene terephthalate) (PET) are widely used cell culture substrates. These polymer membranes are fabricated with pores via soft lithography (PDMS) or track etching (PET and PC) to enable the diffusion of nutrients and signaling molecules when used as support barriers across coculture models.^36,142^ PDMS membranes have been employed as simple scaffolds to mimic the BM due to the ability to vary its elastic properties via manipulating the amount of cross-linker during its fabrication.^38,143−147^ This allows for reducing the modulus of the membrane close to that of the BM.^148^ Huh et al. developed a microfluidic device to mimic the alveolar-capillary barrier with the use of a 10 μm thick and porous PDMS membrane. This membrane was mechanically actuated to induce axial stretch of the PDMS and enabled an artificial breathing motion.^38^ Similarly, the mechanical property of the PDMS membranes was further used by Stucki et al, where an array of alveolar coculture was actuated to stretch via passive perfusion of media through the channels of the chip.^149^ Furthermore, to bring it closer to native BM dimensions, extremely thin PDMS membranes with 2 μm thickness have been fabricated with controlled pore sizes of 3 and 5 μm to enable closer contact between astrocytes and endothelial cells to reconstruct relevant blood-brain barrier models.^150^ Despite the ability to control thickness, pore size, and elastic modulus, PDMS is highly hydrophobic, which makes it difficult to support prolonged cell adhesion. Although surface treatments such as plasma and coating with ECM proteins have enabled their use in supporting cell layers,^151^ these modifications are short-lived and do not allow long-term adhesion of sensitive primary cells.^151^ Moreover, the hydrophobic nature of PDMS also leads to the adsorption of drugs and proteins from the media that can impact cell growth and physiology.^152^ Other forms of porous polymers include PC and PET membranes that have been integrated into the popularly used Transwell inserts where they mimic BM to construct *in vitro* models for the blood-brain barrier, alveolar-capillary barrier (Figure 4), airway models, kidney glomerular, and skin tissue models.^153−160^ The commercially available inserts are flexible in the choice of pore size and can be coated with ECM proteins to promote cell adhesion. However, they do not adequately represent the features of the BM as they lack interconnected porosity, fibrous architecture, and exhibit elastic modulus toward the higher end of the GPa range (Figure 3).^161^ The group of Stone et al. has used such insert systems with 3 μm pore size to mimic the blood-brain barrier BM which supports four different cell types. *In vitro,* alveolar barrier models were also developed and optimized on transwell systems by Hermanns et al.^159^ The group of Kasper et al. improved on these studies and conducted further research regarding the inflammatory and cytotoxic response of these alveolar-capillary models.^162^ However, although basic BM features like the separation of cell monolayers or the study of basic disease models are possible, their mechanical and chemical properties are static and cannot be adapted to a specific *in vivo* BM microenvironment. The impact of the physical microenvironment on various cellular biological processes has been well established and highlights the importance of choosing the right scaffold to investigate *in vitro* organ models.^163^ In our previous work, we showed a comparison of an alveolar-capillary model developed on a thin nanofibrous BM mimic with a conventional PET transwell insert membrane. It was found that the open network structure of the nanofiber mesh allowed a sufficiently direct contact and signal transfer between epithelial and endothelial cells, while in PET membranes the epithelial cell penetrated through the pores and formed a rather imperfect cellular sheet at the endothelial side. However, the porosity of the PET membrane was significantly lower than that of the nanofiber mesh (14% vs 71%).^41^ Another important observed feature was that 94% of CD31+ endothelial cells of the coculture models expressed α-SMA on PET compared with only 28% on the nanofiber membrane, and this was due to the difference in nanofiber topology and stiffness as compared with PET.^33^ In another example, the significance of fibrous architecture on endothelial cell network forming capabilities has been justified further by Davidson et al.^41^ They observed endothelial network formation on Matrigel and electrospun dextran methacrylate as long as cells were able to remodel and recruit the fibers on the respective scaffold. This describes the regulatory effect of mechanical features of an environment on various cellular activities. As a result, we can conclude that PC and PET polymer membranes might be obsolete in the recapitulation of specific organ BM functions. Despite the low production cost, ease of handling, and robust nature of the porous polymeric membranes, they are not entirely appropriate to replace and mimic the complex nature of the BM.

## Hydrogel as BM Mimics

Biocompatible hydrogels embellished with natural BM protein derivatives have been fabricated as a reminiscence of native BM.^166^ Collagen I, laminin, fibrin, alginate, and hyaluronic acid have been used as components to fabricate cellular matrices or hydrogels that are close representatives of the BM environment.^39,167−169^ Additionally, decellularized extracellular matrix (dECM) are emerging cell-free platforms that incorporate the native 3D tissue structure along with inherent bioactive features. These are derived from harvested organs and tissues that are made free of cells or extracted from long-term *in vitro* cell cultures.^170,171^

Some commonly used decellularized ECM-BM replicates commercially available are Matrigel (Corning), Geltrex (Invitrogen), and Cultrex (Trevigen). These are solubilized reconstituted BM extracts derived from Engelbrecht-Holm-Swarm (EHS) sarcoma mouse cells^172^ and are widely used in maintaining organoids and human pluripotent stem cells. Geltrex has been used to maintain and scale up human pluripotent stem cells (hPSC) that can be used for downstream differentiation for therapeutic applications.^173^ These naturally derived matrices have also been used to support and maintain various organoids including intestine, brain, inner ear, prostate, and lung.^174−178^ However, although these matrices are of natural origin, there are limitations to their application as BM mimics such as precise working temperatures (4 °C) to prevent gelling, tuning their biochemical or biomechanical properties without influencing other material attributes,^166,179^ and a lack of knowledge of their molecular composition. These factors tend to limit their translation.^180,181^ However, the group of Mikhail et al. has used Matrigel together with collagen I to produce 3D hydrogels that support mini-gut culture. Collagen I provided mechanical support, and Matrigel offered key components of cell-adhesion present in BM, and when they were used in a perfusable microdevice, they offered support to intestinal stem cells for organoid formation.^182^

The organs from which dECM have been harvested range from skin,^183^ lung,^184^ cornea,^185,186^ bladder,^186^ kidney,^187^ placenta,^188,189^ amniotic membrane,^190−192^ cartilage,^193^ adipose tissue,^194,195^ esophagus,^195^ and liver^196^ and have been used in organ regeneration and *in vitro* model construction. Comercially available human decellularized dermal matrix (Glyaderm) was shown to enhance re-epithelialization and healed full thickness skin defects when seeded with adipose derived stem cells (ASC) in murine wound models.^197^ Similarly, AlloDerm, provided structure and support to biologically engineered blood vessels which were mechanically stronger than vessels lacking AlloDerm ECM.^183^ The group of da Mata Martins et al. demonstrated the importance of choosing appropriate decellularizing techniques to preserve the ultrastructures of decellularized human cornea where the epithelial BM (EBM) was preserved. This intact EBM structure was reported to differentiate the human embryonic stem cells to epithelial-like cells.^185^ The influence of using native-like ECM scaffolds on cell proliferation and differentiation was supported by the work of Sobreiro-Almeida et al.^187^ They fabricated a bioink based on unmodified porcine decellularised kidney ECM that supported the growth of renal progenitor cells which can be used to develop renal *in vitro* tissue models.^187^ Another widely used biomaterial is decellularized placenta, which is rich in growth factors (fibroblast growth factor (FGF), platelet-derived growth factor (PDGF), vascular endothelial growth factor (VEGF), epidermal growth factor (EGF)),^198^ ECM components,^199,200^ and does not require invasive methods to isolate.^199^ Human placenta-derived ECM was reported by Zhang et al. to induce and restore hair growing potential of highly passaged human papillary dermal cells.^201^ Similarly, porous hybrid placental-ECM sponges (PIMS), derived by combining silk fibroin and placental ECM, displayed the potential to regenerate bone tissue.^202^ Apart from placenta, decellularized amniotic membranes (the inner lining of the placenta) have also been exploited due to a rich pool of growth factors and intact BM component.^203^ The group of Nasiry et al. reported the successful use of microporous 3D decellularised amniotic membranes scaffold for wound healing in diabetic rats.^204^ Comparable to the native ultrastructural and molecular properties, the use of human amniotic membranes as scaffolds to support porcine urothelial cells was reported by group of Jerman et al.^205^ Despite the ability to mimic the native tissue architecture and provision of bioactive cell adhesion sites and growth factors, the use of dECM is limited. These include undetected residual toxic substances post decellularization,^206^ degradation rate of the scaffold,^207^ batch to batch variability,^208^ as well as undefined molecular composition.^209^

Another interesting biopolymer which has been widely used to mimic BM is silk fibroin. Silk fibroin is derived from *Bombyx mori* cocoons, and alone or in combination with other polymers, it has been used to produce mechanically stable, biofunctional, degradable, and biocompatible scaffolds.^210^ Due to its versatile nature, silk fibroin has been widely used in tissue engineering including bone,^211^ cartilage,^212^ vascular,^213^ and cancer models.^214^ Stable silk fibroin by enzymatic cross-linking via horse radish peroxidase (HRP) have been employed as 3D mimics to study colorectal cancer cells.^215^

Comparatively, to some extent, synthetic hydrogels bear resemblance to the ECM, due to the presence of polymer networks formed via covalent and noncovalent interactions in the water-swollen environment.^216,217^ Fine-tuning of their bulk mechanical properties by controlling the cross-linking and molecular density favors their use as BM mimics.^218,219^ Frequently used polymers as hydrogels include polyacrylamide (PAAm), poly(ethylene glycol) (PEG), poly(acrylic acid) (PAA). Augmentation of hydrogels with peptides that represent ligands for cell attachment via integrins, protease degradative molecules, and a repertoire of growth factors bring it a step closer to its native counterpart.^220^

Biofunctionalization of hydrogels is achieved via the incorporation of short peptide sequences found in proteins such as collagen and laminin as seen in Figure 5.3. This enhances cellular binding via integrin to the hydrogel mimic.^221^ A commonly used peptide sequence is RGD (Arg-Gly-Asp); however, IKVAV (Iso-Lys-Val-Ala-Val) and YIGSR (Tyr-Ile-Gly-Ser-Arg) are also used along with RGD to tune the cell adhesion property of the gel without affecting the mechanical property of the hydrogel.^222,223^ Additionally, modified 3D silk fibroin hydrogels were covalently linked to IKVAV peptide via EDC/NHS, which promoted neural stem cell differentiation.^224^

**Figure 5:**
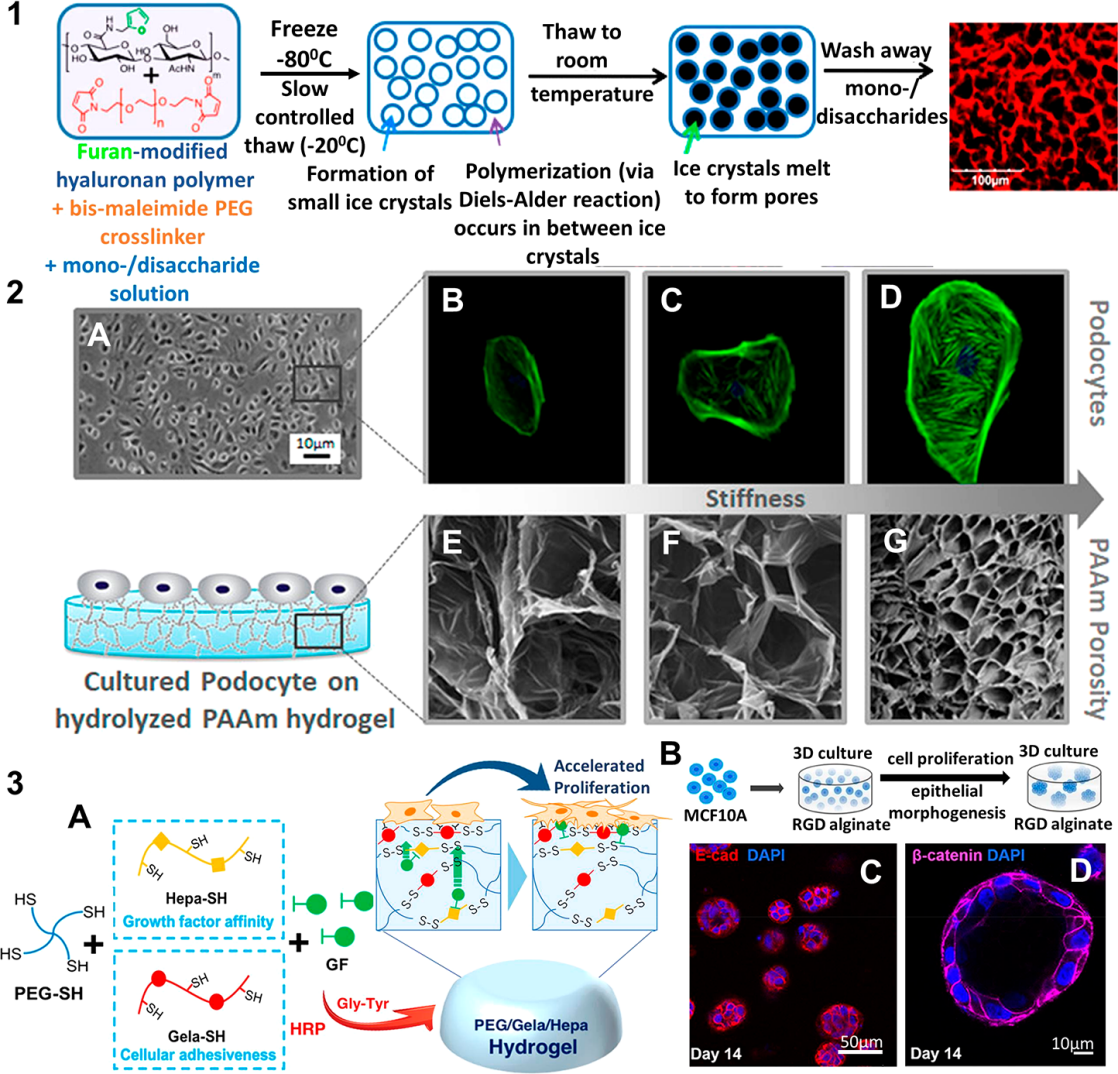
Optimization of hydrogel properties to mimic basement membrane structure and function: Modification of physical and chemical properties of hydrogels have been addressed in varying methods that allow their use as scaffolds to mimic BM: (1) Limitations of pore formation have been overcome using Diels–Alder click chemistry and cryo-gelation of the agarose and hyaluronic acid hydrogels. The polymer mixture is frozen, where the ice crystals slowly melt and are replaced by pores; reprinted with permission from ref (232). Copyright 2017 American Chemical Society; (2) Tunable physical properties of the hydrolyzed polyacrylamide (PAAm) such as stiffness (0.3–300 kPa) and pore size have been exploited to mimic the glomerular filtration barrier with the podocyte cells and also to study the influence of scaffold mechanical properties on such a filtration barrier *in vitro*; reprinted with permission from ref (248). Copyright 2019 American Chemical Society; (3A) Cell adhesion and proliferation on hydrogel BM mimics have been enhanced including the development of biofunctional PEG hydrogels using HRP mediated cross-linking of thiolated polymers, where the 4-arm PEG-SH was conjugated with thiolated gelatin (Gela-SH) and heparin (Hepa-SH); reprinted with permission from ref (249). Copyright 2019 American Chemical Society; (3B) hydrogels are commonly used to realize a 3D environment specifically for proliferation of breast epithelial cells as well as to mimic a tumor environment, where MCF10A cells are embedded in RGD functionalized alginate gels to form (3C) spheroids and (3D) acini like structures similar to *in vivo*; adapted with permission from ref (250). Copyright 2020 Barros de Silva. https://creativecommons.org/licenses/by/4.0/. Changes were made to copyright material.

To mimic vascular BMs, bioactivation of PEG hydrogels by RGD peptides of 20 and 10 kDa was achieved by the group of Gonzalez et al. The hydrogels featured a characteristic elastic modulus (84 and 55 kPa) and pore size (0.16 and 0.19 μm), which is similar to the natural vascular BM.^225^ The influence of native characteristics and bioactivity was observed in favor of the hydrogels in terms of endothelial cell spread and expression of cellular adhesion molecules. On exposure to TNFα (Tumour necrosis factor), cells on hydrogels demonstrated an increased expression of VCAM (vascular cellular adhesion molecule) compared with that on control PC membranes, whereas E-selectin and ICAM (intercellular adhesion molecule) were not significantly different. Additionally, neutrophil capture on the hydrogels was 5 to 7-fold higher than that on stiff PC membranes. These results are similar to a 10-fold increase in neutrophil capture observed *in vivo* after LPS (Lipopolysaccharide) treatment. Furthermore, the study of bone and liver vascularization including the extent and maturity of vascular networks was conducted using 3D BM mimics by the group of Klotz et al.^226^ They developed a hybrid of gelatin cross-linked with synthetic PEG hydrogels as vascular BM mimics. The cross-linking was achieved by factor XIII, where glutamine sequence was incorporated in 8-arm PEG that reacted with the lysine residue present in gelatin. This mimic was fabricated using unmodified cell binding ligands and also enables the incorporation of alternative bioactive compounds containing lysine residues.^226^ Similarly, using the EDC/NHS reaction, cross-linking between PEG, collagen peptide (CLP), and RGD was achieved. The biofunctional cross-linked gels were used for neuronal cell cultures.^227^

Additionally, incorporation of matrix-metalloproteinases (MMPs)-cleavable peptides can impart degradable properties to the hydrogel. This is due to the degradative action of membrane-bound or cell-secreted enzymes, namely MMPs that are involved in ECM remodeling. Numerous cellular processes including proliferation and migration occur during the remodeling process to establish tissue homeostasis. The presence of MMP cleavable peptides in the hydrogels activates the degradation cascade of the BM mimic and also offers partial or complete replacement of the synthetic BM by cellular ECM deposits.^228,229^ Incorporation of the peptide sequence recognized by membrane-type matrix metalloproteinase-1 (MT1-MMP) was achieved by the group of Ricardo et al.^230^ The synthetic hydrogel was fabricated using four-armed maleimide-terminated PEG incorporated with cell adhesive peptides RGD as well as MT1-MMP degradable peptide sequences via Michael-type addition reaction. These functionalized and cross-linked hydrogels provided an optimal microenvironment and were reported to initiate renal epithelial tubulogenesis of the inner medullary collecting duct cells.^230^ Such existing synthetic functionalized BM hydrogels with MMP-cleavable sequences provide platforms that can be used to understand and study remodeling processes involved in tissue repair, immune cell migration, and cancer metastasis. Moreover, controlled degradation alters the mechanical properties of the hydrogel which can further be exploited to activate cellular processes affected by substrate rigidity such as stem cell differentiation.^231^

Another aspect is the ability to artificially enrich the synthetic hydrogels with growth factors that regulate the wide array of cellular interaction and behavior analogous to the native BM microenvironment.^220^ This is observed in hybrid agarose-hyaluronic acid hydrogels that allowed controlled spatial and gradient immobilization of biomolecules using photosensitive molecules via Diels–Alder click chemistry, which was developed by the group of Tam et al.^232^ Two-photon irradiation exposed reactive sites to biomolecules present in the hydrogels and allowed investigation of endothelial cells exposed to a concentration gradient of modified vascular endothelial growth factor (VEGF-165). Additionally, these BM mimics allowed the embedding of MMP-cleavable peptides in the 3D hydrogel system offering the possibility to analyze metastatic migration through the hydrogels.

Cell-laden 3D hydrogels can be used to mimic complex tissue structures using printing techniques such as microextrusion, laser-assisted, inkjet, and stereolithography.^233,234^ The group of Puperi et al. developed 3D PEG hydrogel based systems to recapitulate endothelialized aortic valve models by maintaining a coculture of vascular interstitial cells (VIC) and vascular endothelial cells (VEC).^235^ Spatially controlled introduction of cell type-specific ligands in PEG hydrogels enabled the distribution of endothelial cells to the periphery and interstitial cells to the center, which is similar to the physiological distribution of cells in heart valves. This study could demonstrate that anisotropic cell type-specific ligand distribution can be used to control cell position in 3D matrices in order to study healthy and diseased conditions *in vitro* including cell metastasis, atherosclerosis, and drug response. To overcome shear stresses induced on cells during extrusion printing, a new method of digital light processing (DLP), which uses photopolymerization to print layer by layer 3D structures using cell embedded hydrogels. A 3D hydrogel combining silk fibroin and polyethylene glycol acrylate (PEG4A) was reported to maintain and proliferate human primary keratinocytes at an air–liquid interface.^236^ Similarly, silk fibroin and glycidal methacrylate bioink developed by the groups of Kim et al. was used in DLP to produce precise scaffolds that can mimic tissues including heart, vessel, brain trachea, and ear.^237^

The benefits of using synthetic hydrogels were further justified by the ability to manipulate mechanical properties compared with conventional collagen gels to study various aspects of valve disease conditions.^235^ The group of Contessi Negrini et al. established mechanically tunable 3D gelatin hydrogels modified using tetrazine and norbornene via biorthogonal click chemistry. By tuning the ratio of tetrazine to norbornene and their degree of modification, hydrogels ranging from 1 to 5 kPa were achieved that were used to embed human dental pulp stem cells.^238^

Moreover, the ability to manipulate shapes of synthetic PEG-diacrylate (PEGDA) hydrogels to mimic curved environments for ducts and acini of mammary glands presents the versatility and high level of control achievable with synthetic materials in the field of biomimetics. The curvature was achieved by using different molecular weight bilayers of PEGDA that swelled at different ratios by releasing from the underlying substrate. This together in combination with photopatterning provides another opportunity in the production of varying structures of ducts and acini, while additional cross-linking of PEGDA with gelatin methacrylate increases cell adherence to the substrate.^239^ Freedom to manipulate hydrogel shape allows close resemblance to physiological structures that influences cell response and behavior *in vitro*.

Although the use of synthetic hydrogels offers the possibility to tune a broad spectrum of material properties such as stiffness, porosity, shape, and the spatially controlled incorporation of adhesive ligands and degradable sequences, hydrogel BM mimics still lack certain key characteristics found in native BMs. For example, the minimal achievable free-standing hydrogel thickness lies to date between 10 and 13 μm, which is at least 10 times thicker than native BMs.^225,240^ This difference in scaffold thickness combined with the soft nature of hydrogels renders meaningful cocultivation of different cell types on opposing scaffold sides extremely challenging.^42,240−242^ While the separation distance between cell layers is far too large for direct cellular communication, the softness of the material prevents cell cultivation on opposite sides of free-standing hydrogel layers.^242^ However, these limitations can be overcome using support structures such as meshes and cross-linking chemistry to improve hydrogel stability.^240,243,244^ The group of Zamprogno et al. was successful in establishing an alveolar-capillary coculture model on a ∼10 μm thick collagen-elastin supported on a gold mesh.^240^ 3D gelatin methacrylolyl (GelMA) hydrogel scaffolds were designed to mimic lung alveoli structures to support alveolar cells on a less than 3 mm thick hydrogel at air–liquid interface.^243^ Despite thickness limitations and lack of fibrous structure, hydrogels are popularly used to mimic the intricate 3D microenvironment of tissues using printing approaches and photopatterning of cell-laden gels.^245,246^ Successful 3D structures in microfluidic chips have been achieved by photopatterning of cell-laden gelatin hydrogels (Table 3).^247^

**Table 3:** Biomimetic Hydrogel Scaffolds Reproduced via Decelluarized ECM, Synthetic or Natural Polymers to Replicate Tissue Microenvironments. The Table Describes the Synthesis of Hydrogels Using Different Chemical Approaches^a^

polymer	preparation method	pore size [μm], scaffold thickness [μm]	biofunctional component	cell line	tissue model	application	ref
gelatin14%\HA tyramine 1% w/v	spin coating	-, 15.5 μm	KGF, FGF	Calu-3, A549, human MSC	respiratory epithelium	ECM mimic for MSC-derived epithelial patches	(251)
chitosan\dextran	Michael addition reaction	5–20 μm, -	FGF	NIH3T3	-	ECM mimics for wound healing	(252)
PEG-8SH\TEDVE	thioester exchange	-, -	GRGDS	human MSC	-	ECM mimics for MSC	(253)
GelMA	photoinitiated polymerization	-, 1000–2000 μm	-	human articular chondrocytes, human ECFC, human MSC, human OV-MZ-6	cancer models	3D ECM mimics	(254)
PEG diacrylate	-	-, 150 μm	collagen IV	human RPTEC, NHLF	acute kidney injury model	ECM mimics for renal epithelial cells	(255)
BPAA\diphenylalanine	π–π stacking	-, -	-	L929	-	ECM mimic	(256)
PNIPAM	radical polymerization	-, -	-	3T3-L1, HEK293, A549	-	ECM mimic	(257)
collagen\alginate\fibrin	hydrogen bond formation	40–120 μm, -	-	L929, MIN6, Y201 hMSC	musculoskeletal, pancreatic models	ECM mimic for soft tissues	(258)
collagen\laminin\HA	hydrogen bond formation	-, -	-	ratNPC	spinal cord injury	ECM mimic for neural tissue engineering	(259)
pLysAAm\HA	photopolymerization	150 μm, -	-	MCF-7	breast tumor	mimic breast tumor microenvironment	(260)
tetrazine and norbornene modified gelatin	biorthogonal cross-linking	-, -	-	hDPSC	3D hydrogels	compartmentalized cocultures	(238)
PEG	EDC/NHS	-, 500 μm	collagen peptide, RGD	rat neuron, rat astrocytes	cerebellar cell cultures	functional organoids	(227)
gelatin, alginate	Michael addition reaction, ionic reaction	400 μm, -	-	hMSC	-	adipose tissue engineering	(261)

a Keratinocyte growth factor (KGF), fibroblast growth factor (FGF), hyaluronic acid (HA), mesenchymal stem cells (MSC),eight arm-poly(ethylene glycol) (PEG-8SH),thioester di(vinyl ether) (TEDVE), gelatin methacryloyl (GelMA), 4-biphenylacetic acid (BPAA), poly-*N*-isopropylacrylamide (PNIPAM), collagen alginate and fibrin (CAF), poly-*N*-acryloyl l-lysine (pLysAAm), human lung adenocarcinoma cell line (Calu-3), human lung carcinoma epithelial cells (A549), mesenchymal stem cells (MSC), endothelial colony forming cells (ECFC), ovarian cancer cell (OV-MZ-6), renal proximal tubular epithelial cells (RPTEC), normal human lung fibroblast (NHLF), mouse fibroblasts (L929), mouse preadipocyte cells (3T3-L1), human embryonic kidney cells (HEK293), mouse pancreatic β cells (MIN-6), human TERT mesenchymal stem cells (Y201 hMSC), neural progenitor cells (NPC), human breast cancer cell line (MCF-7), human dental pulp stem cells (hDPSC).

## Electrospun BM Mimics

Electrospinning is a versatile technique that enables the production of micro to nanofibrous scaffolds that can be modulated in terms of their surface morphology, fiber diameter size, mesh thickness, and fiber arrangement.^262^ Multiple synthetic polymers ranging from poly(caprolactone) (PCL), poly(lactic acid) (PLA), poly(glycolic acid) (PGA), poly(lactic-*co*-glycolic acid) (PLGA), as well as polymers of natural origin including chitin, collagen, gelatin, and their hybrids have been utilized to produce diverse electrospun scaffolds in the field of tissue engineering.^263^

The potential of achieving desired mechanical (pore size, fiber diameter, topology) and chemical (functionalization) properties by varying electrospinning parameters, including polymer, solvent, flow rate, distance, voltage supply, and so on, has been exploited to tailor BM mimics. Many such *in vitro* models include tissue models of skin, glomerular filtration units as well as alveolar-capillary barrier models.^43−48^ A natural polymer, laminin I, extracted from murine has been electrospun to produce nanofibrous scaffolds that mimic BM in aspects of morphology including fiber diameter, pore size as well as architecture. Unlike electrospun biopolymers including collagen and fibronectin, laminin does not require cross-linking modification steps to maintain its fibrous morphology when exposed to an aqueous environment.^264,265^ The group of Neal et al. fabricated electrospun laminin I nanofibers without cross-linking modification. The scaffold maintained a fibrous morphology even after exposure to cell culture media.^266^ This property was attributed to the use of lyophilized laminin, which is considered to be insoluble in aqueous media and also to possible structural changes in the protein during elctrospinning that make the laminin insoluble in an aqueous media.^267,268^ However, use of laminin nanofibers as an alternative platform to mimic BM is still limited. Since commercially available laminin is majorly derived from human placenta and exhibit batch-to-batch variability. Moreover, compared to other ECM proteins, laminin is expensive due to difficulties in obtaining sufficient yields of its active form. Thus, it is mainly used to culture specialized cells like neuronal stem cells.^269^

Silk fibroin, alone or in combination with other natural polymers has been used to produce mechanically stable and biocompatible nanofibrous scaffolds.^270,271^ Electrospun silk fibroin has been used successfully to support many tissue cells including cartilage,^272^ mucosal cells,^273^ bone,^274^ endothelial,^275^ and nerve cells.^276^ Electrospun silk fibroin nanofibers fabricated by Mou et al. (Figure 6.1) were successfully used to mimic glomerular BM, which supported the proliferation and differentiation of human podocytes.^277^ Additionally, a hybrid silk fibroin and chitin nanofibers were incorporated with TGF-β, to support the adhesion and proliferation of chondrocytes.^278^ Apart from bone tissues, electrospun silk fibroin has also been used to produce stable vascular grafts that proliferate endothelial cell growth.^279^ In addition, laminin-coated electrospun silk fibroin mats have been promising tools for proliferation and differentiation of neural progenitor cells.^280^ Moreover, a combination of electrospinning and microfluidics has been used to produce layer by layer pure silk nanofibers and their microdroplets to sustain endothelial cell monolayers and prevent thrombus formation.^281^

**Figure 6:**
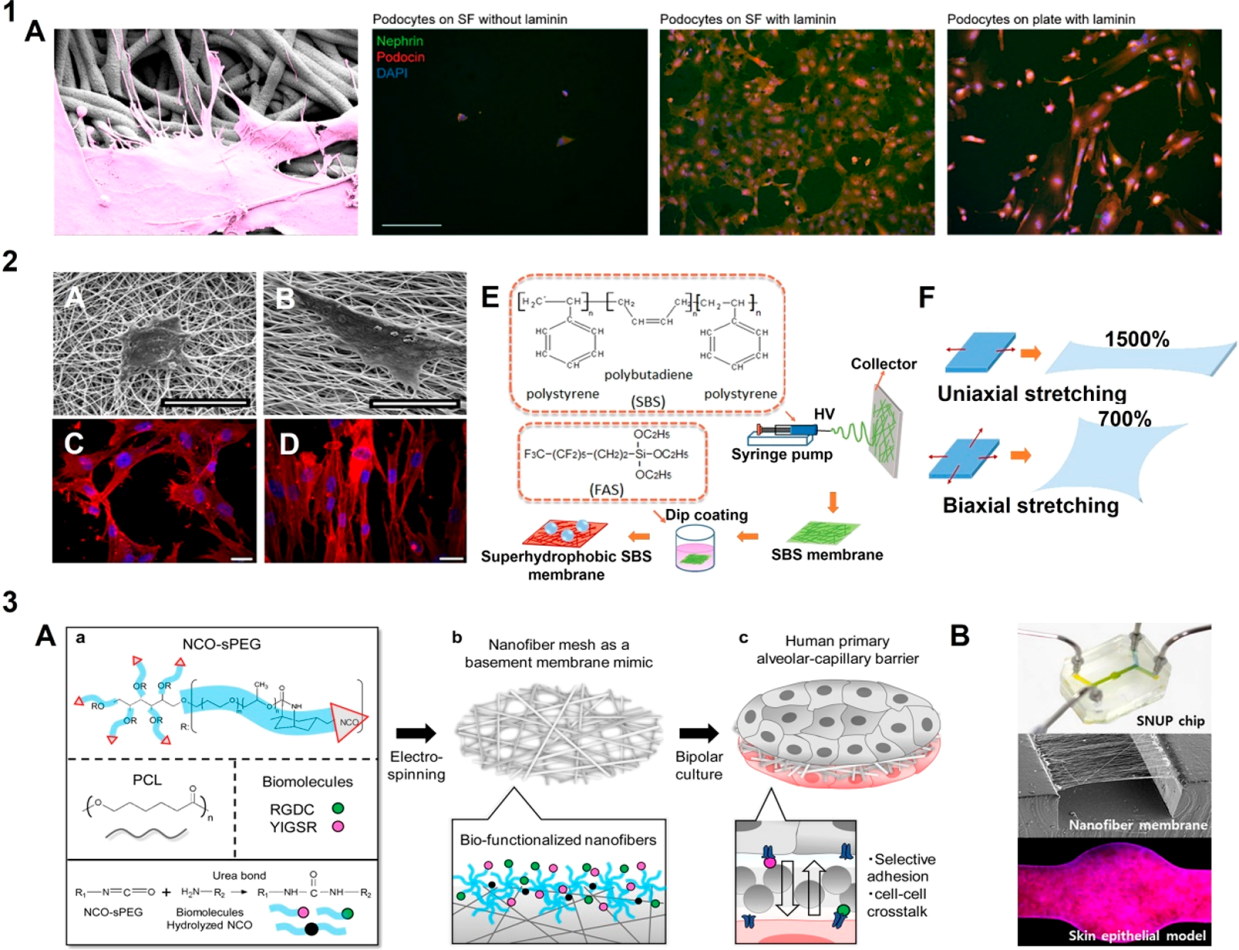
Fibrous basement membrane mimics: Electrospinning is a versatile technique to produce fibrous scaffolds of varying properties in order to mimic the native BM in terms of architecture strength and dimensions of the fibers: (1A) A laminin-coated nanofibrous membrane of silk fibroin (SF) was fabricated as glomerular BM mimic, for differentiation of human podocytes from human stem cells, where (A) a podocyte interacts with the laminin coated SF fibers and confocal images of podocytes stained for podocin (red), nephrin (green) and nucleus (blue), on SF without laminin, SF with laminin and on tissue culture plate; adapted with permission from ref (277). Copyright 2022 Mou, Xingrui et al. https://creativecommons.org/licenses/by/4.0/ (changes were made). (2) Varying mechanical and topographical properties is possible where (A–D) random and aligned forms of PCL-gelatin meshes are exploited to mimic specific BM, where NIH3T3 cells are shown to respond respectively by cellular spread or elongation; adapted with permission from ref (295). Copyright 2016 Fee et al. https://creativecommons.org/licenses/by/4.0/ (changes were made). (E,F) optimization of elastic modulus can be achieved where superhydrophobic and elastic fibers were fabricated by dip coating poly(styrene–butadiene-styrene) (PBS) fibers in fluoroalkyl silane (FAS) to produce fibers that can be stretched both uniaxially (1500%) and biaxially (700%) even after 1000 stretch cycles; adapted with permission from ref (296). Copyright 2015 Hua Zhou et al. http://creativecommons.org/licenses/by/4.0/ (changes were made). (3) Electrospinning allows biofunctionalization as seen in (A) synthetic alveolar-capillary BM for *in vitro* expansion and study of pulmonary cells, where electrospinning of PCL and the surface segregated isocynate end groups of six-armed sPEG form covalent bonds with the amine groups of bioactive peptides via urea bond formation; reprinted with permission from ref (41). Copyright 2017 American Chemical Society (no changes); (B) Electrospun fibers can also be used as free-standing membranes in microfluidic chips to analyze effect of dynamic shear on cells; reprinted with permission from ref (297). Copyright 2021 American Chemical Society (changes were made).

The use of hybrid blends of both natural and synthetic polymers to fabricate a scaffold has been frequently considered. For example, decellularized kidney ECM from porcine and PCL blend was electrospun to fabricate kidney filtration BM mimic by Sobreiro-Almeida et al.^282^ They concluded that the high content of ECM in the polymer blend aided in closer representation of the renal BM and enhanced cell line biological activity such as adhesion, proliferation, and migration as well as the formation of tight junctions compared to pure synthetic PCL scaffolds. Another group of Slater and colleagues have also successfully developed a trilayer glomerular filtration model that includes an electrospun layer of collagen I and PCL blend that is physically supported on a micro photoelectroformed (PEF) nickel mesh to recapitulate the glomerular BM. This artificial BM is fixed onto a cell crown followed by coculture of immortalized cell lines of GEnC (glomerular endothelial cells) and podocytes on opposite sides of it. They were able to demonstrate a monolayer formation of GEnC and a semimonolayer formation of the podocyte cell layers on the opposite sides of the BM mimic.^34^

Moreover, the potential of electrospun fibers to maintain cell growth and prevent cell infiltration was proposed by the group of Bye et.al. They recapitulated skin BM by fabrication of a triple-layered electrospun scaffold composed of nanoporous poly hydroxybutyrate-*co*-hydroxyvalerate nanofibers sandwiched between two layers of microporous poly L-lactic acid microfibers. This 3D scaffold not only allowed adherence and proliferation of keratinocytes and fibroblast cell layers on the opposite sides but also impeded cell infiltration. Despite the physical impedance to the movement of cells, the formation of a developed epithelium indicates the ability of keratinocytes and fibroblast cell layers to communicate with each other across the BM mimic.^283^

Scaffolds comprised of pure synthetic polymers have also been adapted to support cell adhesion and proliferation. Electrospinning permits conjugation of cell adhesive sequences to obtain biofunctionalized scaffolds that enable efficient and long-term adhesion of primary cells in particular.^262,284^ The group of Mollet et al. established a synthetic BM mimic by electrospinning ureidopyrimidinone (UPy)-PCL polymers and functionalized it with UPy-peptides. The hierarchical arrangement of the fibrous micro and nanofibers of UPy -PCL along with the presence of a customized bioreactor accentuated its likeness to the naturally occurring BM environment of the renal tubule epithelial cells. Human kidney 2 (HK2) cells were observed to proliferate on the freestanding mimics under both static and dynamic conditions maintained in customized bioreactors that allowed separate media flow on the apical and basal side of the cells.^40^

Furthermore, evidence of the use of biofunctionalized electrospun fibers as biomimetic BM in the establishment of a bipolar coculture model of alveolar-capillary barrier (Figure 6.3) is observed in the works of Nishiguchi et al. A 10 μm thick mesh was fabricated via electrospinning of PCL and bioinert six-armed, star-shaped poly(ethylene oxide-*stat*-propylene oxide) with isocyanate end groups (NCO-sPEG) and functionalized via short RGD peptide sequences. Resembling the BM of the alveolar-capillary barrier, the electrospun mimic provided a scaffold where human primary pulmonary alveolar epithelial cells (HPAEC) and human umbilical vein endothelial cells (HUVEC) were successfully proliferated as monolayers on opposite sides as a bipolar culture. This work also portrayed the eccentric behavior of the HPAEC infiltration to the HUVEC layer when seeded on PC membranes of commercially available transwell inserts.^41^

Apart from obtaining biofunctionalized scaffolds as an important criteria for cell adhesion, the cells often respond to the mechanical and topographical features of substrates as well. The significance of scaffold thickness, porosity, and fibrous architecture on the formation of the functional alveolar-capillary barrier was highlighted by Jain et.al. by comparing ultrathin 2 μm electrospun nonwoven PCL meshes with commercial 10 μm thick PET membranes.^33^ The 21 days’ stable coculture model demonstrated integral barrier formation and the absence of cell layer infiltration despite the highly porous and ultrathin nature of the PCL meshes. Interestingly, these models displayed a similar response as *in vivo* when induced with inflammation using IL8 where the neutrophils transmigrated across the double cell layers and the BM mimic to reach the site of cytokine addition. This highlights that universally required porous polymeric membranes with 3 μm pores or large can be replaced by electrospun fibers for such investigations (Figure 4.3). Besides demonstrating successful neutrophil migration, the endothelial cells expressed a higher percentage of mesenchymal marker αSMA (smooth muscle actin) on PET membranes compared with that on PCL meshes, which further supports the influence of scaffold on cell behavior.

Moreover, electrospinning of synthetic polymers also allows the incorporation of bioactive molecules including MMP-cleavable sequences to fabricate BM mimics with controllable mechanical and physical properties. Kim et al. fabricated nanofibers that released DNA linked to the fibers by MMP cleavable sequence in response to high MMP presence during diabetic ulcers.^285^ This was achieved by using a PCL–PEG block copolymer that had surface-exposed amine groups. The MMP cleavable sequence was linked to the fiber via amine groups and the linear polyethelineimine (PEI) was chemically conjugated to the MMP sequence. Due to electrostatic interaction, the DNA molecules bound to the linear PEI and DNA release were confirmed in the presence of MMP. Despite the ability to chemically modify, these synthetic mimics lack water retention and flexibility to an extent compared with the natural ECM and hydrogels. This limitation was addressed by electrospinning of methacrylated hyaluronic acid along with a photoinitiator and carrier polymer to produce photo-cross-linkable fibrous hydrogels.^286^ Similarly, electro-conductive nanofibers were fabricated using a blend of gelatin-polyaniline, and a blend of hydrogel gelatin and 4-armed PEG, using novel cross-linking chemistry, to mimic retinal BM.^287^

Furthermore, improvements by incorporation of protease-sensitive motifs in electrospun hyaluronic acid were carried out by Wade et al.^288^ Michael addition reaction between maleimides and thiols was exploited by using hyaluronic acid modified with maleimide and methacrylated cleavable peptides. Moreover, other cell adhesive peptides can also be embedded in these fibrous scaffolds. This allows the production of electrospun hydrogels that closely resemble the native BM with respect to fibrous architecture as well as the presence of bioactive molecules that play a major role in cell behavior and response.

Although synthetic electrospun scaffolds are ideal platforms to mimic BM, they are unable to completely resemble the 3D complexity of the natural form which is responsible for regulating various cellular functions.^56,289^ Electrospinning can be combined with other techniques including the manual folding and unfolding of nanofiber mesh where 3D electrospun nanofibers can be achieved by stacking layers of cell laden nanofibers or centrifugal electrospinning.^290,291^ However, these techniques are limited due to operator skill as well as limited fiber morphology using centrifugal electrospinning. The mechanical stability offered by fibrous electrospun scaffolds allows their use as freestanding substrates for 2.5D cocultures on opposite sides of the BM mimic seen in alveolar-capillary barrier models supported on electrospun biofunctionalized PCL membranes^41,292^ and in microfluidic devices that represent *in vitro* glomerular filtration models.^40^ However, electrospinning is limited in terms of reproducibility due to high dependence on environmental factors such as humidity and temperature.^293^ The choice of solvents and high voltage used during electrospinning can also lead to loss of bioactivity of biomolecules (Table 4).^294^

**Table 4:** Biomimetic Scaffolds Reproduced via Electrospinning Using Natural, Synthetic Polymers, or a Combination of Both in Various Fields of Tissue Engineering to Obtain Close Representatives of the BM. The Table Describes Various Electrospun Mimics with Their Characteristic Parameters and Morphological Properties along with Biofunctional Components^a^

		spinning properties						
polymer	solvent	flow rate [mL/h], collecting distance [cm], voltage [kV]	fiber diameter [nm], porosity [%], scaffold thickness [μm]	biofunctional component	cell line	tissue model	application	ref
laminin I	HFIP	1.5 mL/h, 12.5 cm, 20 kV	142 nm, -, -	-	hASC	-	BM mimic for neural tissue engineering	(298)
PCL	HFIP	0.5 mL/h, 15 cm, 18 kV	400 nm, -, -	porcine dKECM	HK-2	-	renal filtration barrier	(282)
PET	TFA	1 mL/h, 15 cm, 15 kV	200–600 nm, 83%, 35 μm	gelatin	hEC	vascular graft	blood vessel tissue engineering	(299)
PCL\SF	HFIP	0.2–1.5 mL/h, 8.5 cm, 20 kV	146 nm, -, -	porcine BM extracts	primary epithelial cell	-	esophageal tissue engineering	(300)
PLGA	acetone	0.5 mL/h, 16 cm, 13 kV	300 nm, -, 20 μm	peptides derived from fibronectin, laminin, collagen IV	HF, HaCaT,	skin model	BM mimics for long-term cell coculture.	(301)
SF\PEO	water	1.2 mL/h, 14 cm, 12 kV	267 nm, 54%, -	laminin	ratSC	-	peripheral nerve tissue engineering	(302)
PCL	MC:DMF (80:20)	1 mL/h, 12 cm, 13 kV	135–1095 nm, -, -	Matrigel	C17.2	-	nerve tissue engineering	(303)
PLLA	CF:DMF (4:1 v/v)	0.2 mL/h, 18 cm, 22 kV	1380 nm, 52%, 100 μm	ASA, AM lysate	HUVEC	-	endothelial BM	(304)
PLGA	HFIP	0.36 mL/h, 15 cm, 14 kV	308 nm, -, -	elastin	SIMS	-	salivary gland BM	(305)
4%PEG, 96%PCL	TFE	1 mL/h, 10 cm, 10 kV	500 nm, -, 6 μm	collagen I	TIME, human astrocyte, human pericyte	-	blood brain barrier	(306)
PCL	HFIP	0.35 mL/h, 15 cm, 21 kV	260 nm-350 nm,-,2 μm	collagen I, fibronectin, laminin	HPMEC, NCI-H441	-	alveolar-capillary barrier	(33)
PCL	HFIP	0.5 mL/h, 15 cm, 20 kV	300 nm, -, 10 μm	peptides derived from fibronectin	HUVEC, HPMEC, NCI-H441, HPAEC	-	alveolar-capillary barrier	(41)
PCL	HFIP	1.2 mL/h, 12 cm, 18.5 kV	220–340 nm, -, -	ureido pyrimidinone peptide functionalized	HK-2	-	kidney glomerular filtration barrier	(40)
gelatin-polyaniline and gelatin 4-arm PEG	acetic acid water, NMP	1.5 mL/h, 9 cm, 23 kV	154 nm, -, 4000 μm	-	hRPE	retinal Bruchs membrane	age-related macular degeneration	(287)

a 1,1,1,3,3,3-hexaflouro-2-propanol (HFIP), decellularized kidney extracellular matrix (dKECM), poly-L-lactic acid (PLLA), acetlysalicylic acid (ASA), amniotic membrane (AM),poly lactic-co-glycolic acid (PLGA), 2,2,2-trifluoroethanol (TFE), poly ethyleneglycol (PEG), poly(ε-caprolactone) (PCL), *N*-methyl-2-pyrrolidone (NMP), human adipose stem cells (hASC), human kidney cell line (HK-2), human coronary artery endothelial cells (hEC), human dermal fibroblast (HF), human keratinocyte cell line (HaCaT), SC (Schwann cells), neonatal mouse cerebellum stem cells (C17.2), human umbilicial vein endothelial cells (HUVEC), mouse ductal submandibular epithelial cells (SIMS), telomerase immortalized microvascular endothelial cells (TIME), human pulmonary microvascular endothelial cells (HPMEC), human lung adenocarcinoma epithelial cell lines (NCI-H441, human primary pulmonary alveolar epithelial cells (HPAEC), human kidney cell lines (HK-2), human retinal pigmented epithelium (hRPE).

## Conclusion and Future Perspectives

BM is a vital form of ECM that offers physical support; divides tissues into distinct regions; and provides cues for cellular differentiation, proliferation, and transmigration. The unique structure and composition of the BM at different anatomical locations necessitates the fabrication of mimics that can be tailored to accurately represent specific organ models.^55,56^ However, the use of naturally derived materials as BM mimics is challenging considering their lot-to-lot variability and difficulty in amending their respective biophysical and biochemical properties.^166,179^

Porous and nonporous polymeric membranes have been widely used to replace natural BM in *in vitro* models due to the ease of handling and mechanical robustness (Figure 4). Although these membranes display some key features such as nano- or microporosity, their simplicity in terms of topography and biochemical cues are far from representing the native BM.

Another major synthetic substitute for mimicking the BM are hydrogels. These scaffolds are highly modifiable and allow the adjustment of nanoporosity as well as bulk mechanical properties by cross-linking processes. Additionally, bioactive compounds can be incorporated into the hydrogel, which enables cellular remodeling and provides vital biochemical cues such as adhesive motifs or the spatiotemporal release of growth factors. Despite their lack of fibrous architecture, cell-laden hydrogels aided by 3D printing techniques have been employed to mimic and construct complex tissue microenvironments.^234^

Additionally, electrospinning has been widely and successfully used in the development of synthetic and hybrid BM, which due to their mechanical stability, indirectly support cocultures while accurately incorporating the dimensions of natural BM.^34,40,41,283,301,307^ The versatility of electrospinning with regard to the source material and the collection method enables the optimization of topographical, biomechanical, and biochemical scaffold properties of the ultimate product.^41,262^ As a result, electrospun membranes can be tailored precisely to a specific BM region, while offering additional perks such as defined fiber diameter, topographical guidance, adhesive and degradable motifs.^284^ Moreover, the fabrication of scaffolds from stimuli-responsive polymers can provide additional benefits exceeding the capabilities of natural BM, where cellular actuation is induced by external stimuli including pH, temperature, electrical current and light.^308^

The importance of the choice of scaffold depends on the extent of physical and chemical stability required to mimic the respective organ or tissue system, resources available as well as cellular responses.^32−34^ It is critical to elucidate native cell–BM interaction and improve the fabrication of *in vitro* models as reliable constructs in regenerative medicine. Stable scaffolds for such reproducible *in vitro* models can be achieved by combining the tunable physical and mechanical properties of synthetic polymers together with the biochemical cues of natural polymers.^309−311^ Furthermore, dynamic gradients in stiffness can be created at the cell matrix interface to mimic BM in health and disease conditions.^312,313^

However, vital questions such as the extent of resemblance in terms of composition and mechanical, structural as well as topographical cues to mimic the BM are still unanswered. The BM is constantly evolving and unique at various organ locations in terms of architecture, composition, and functionality during both physiological and pathological events. There is an existential gap of knowledge that can be filled by implementing novel techniques such as computational modeling, imaging techniques, and gene sequencing. Advances in imaging techniques such as 4D imaging and noninvasive mechanical measurements including ultrasound, optical coherence tomography, and magnetic resonance elastography can be exploited to gain insights into the native BM.^212,314,315^ Our understanding of answers to such predominant questions would enable the construction of scaffolds to maintain the stability and functionality of *in vitro* models. The inputs gained from these novel techniques can be integrated to develop simulated models of representative BM, which can further be used as guides to design scaffolds that can realize native properties in the *in vitro* models. This could open doorways to construct reliable mimics of BM tailored to support *in vitro* models in the field of tissue engineering and regenerative medicine.
